# Experimental Study on the Partial Discharge Characteristics of Palm Oil and Coconut Oil Based Al_2_O_3_ Nanofluids in the Presence of Sodium Dodecyl Sulfate

**DOI:** 10.3390/nano11030786

**Published:** 2021-03-19

**Authors:** Nur Aqilah Mohamad, Norhafiz Azis, Jasronita Jasni, Mohd Zainal Abidin Ab. Kadir, Robiah Yunus, Zaini Yaakub

**Affiliations:** 1 Advanced Lightning, Power and Energy Research Centre (ALPER), Department of Electrical and Electronics Engineering, Universiti Putra Malaysia, Serdang 43400, Selangor, Malaysia; jas@upm.edu.my (J.J.); mzk@upm.edu.my (M.Z.A.A.K.); 2 Department of Electrical Engineering, Politeknik Mukah, Mukah 96400, Sarawak, Malaysia; 3 Institute of Advanced Technology (ITMA), Universiti Putra Malaysia, Serdang 43400, Selangor, Malaysia; 4 Department of Chemical and Environmental Engineering, Faculty of Engineering, Universiti Putra Malaysia, Serdang 43400, Selangor, Malaysia; robiah@upm.edu.my; 5 Hyrax Oil Sdn. Bhd., Lot 4937 Batu 5 1/2, Jalan Meru, Mukim Kapar, Klang 41050, Selangor, Malaysia; zaini@hyraxoil.com

**Keywords:** partial discharge, PDIV, palm oil, coconut oil, Al_2_O_3_, nanofluids, SDS

## Abstract

This experimental study aims to examine the partial discharge (PD) properties of palm oil and coconut oil (CO) based aluminum oxide (Al_2_O_3_) nanofluids with and without surfactants. The type of surfactant used in this study was sodium dodecyl sulfate (SDS). The volume concentrations range of Al_2_O_3_ dispersed in oil samples was varied from 0.001% to 0.05%. The ratio of surfactants to nanoparticles was set to 1:2. In total, two different types of refined, bleached and deodorized palm oil (RBDPO) and one type of CO were measured for PD. Mineral oil (MO) was also examined for comparison purpose. PDIV measurements for all samples were carried out based on rising voltage method whereby a needle-sphere electrode configuration with a gap distance of 50 mm was chosen in this study. Al_2_O_3_ improves the PDIVs of RBDPO, CO and MO whereby the highest improvements of PDIVs are 34%, 39.3% and 27%. The PD amplitude and repetition rate of RBDPO improve by 38% and 81% while for CO, it can increase up to 65% and 80% respectively. The improvement of PD amplitude and repetition rate for MO are 18% and 95%, regardless with and without SDS. Without SDS, the presence of Al_2_O_3_ could cause 26%, 75% and 65% reductions of the average emission of light signals for RBDPOA, RBDPOB and CO with the improvement of PD characteristics but both events do not correlate at the same volume concentration of Al_2_O_3_. On the other hand, the average emission of light signal levels of the oils increases with the introduction of SDS. The emission of light signal in MO does not correlate with the PD characteristics improvement either with or without SDS.

## 1. Introduction

Partial discharge (PD) is an event that exists in dielectric fluid or solid insulations. Generally, PD occurs due to the inappropriate design, defect in manufacturing and contaminations either from internal or external sources. PD is usually initiated at conductor-dielectric interfaces within solid or dielectric insulating fluids, bubbles within insulation fluids and particle contaminants in insulation fluids. Materials defect could also enhance local electric field and lead to the inception of PD [1,2]. Once the discharge is initiated, it can accelerate the degradation of insulation materials, allowing its propagation from local areas into bulk oils, leading to breakdowns [3]. The discharge induced insulation failures develop from discharge inception to breakdown depends on the location of the local field and operating condition of transformers [1]. Discharge in dielectric insulating fluids can take a long time to develop. Dielectric insulating fluids should be able to handle high electrical stress without the initiation of PD activities [4]. The partial discharge inception voltage (PDIV) test is one of the most important parameters to determine the condition and intrinsic characteristics of dielectric insulating fluids [1,5]. PD measurement is known as a non-destructive approach that can be used to determine the integrity of dielectric insulating fluids [6].

Nowadays, nanotechnology provides exciting new possibilities especially on the enhancement of the dielectric insulating fluids performances such as electrical properties, PD, diffusivity, thermal conductivity, cooling properties, viscosity, heat exchanger, friction and heat transfer coefficients [7,8,9,10,11,12,13,14,15]. Various factors such as intrinsic properties, volume concentrations, contact areas of solid-liquid, surfactants suspension and synthesis approaches could affect the performances of nanoparticles in dielectric insulating fluids [16,17].

Various studies have been carried out to examine the PD characteristics and PDIV of either mineral oil (MO) or vegetable oil based nanofluids. Different types of nanoparticles such as TiO_2_, Fe_3_O_4_, SiO_2_, silica and fullerene have been previously examined [18,19,20,21,22,23,24,25,26]. Recent studies show that silica and fullerene could improve the PDIV of MO by 25% and 20% under positive DC voltage [18]. Similar positive effect on the PDIV of MO under AC voltage is found as the TiO_2_ is introduced with the percentage of improvement is around 8.2% [19]. Under AC voltage, the introduction of Fe_3_O_4_ and TiO_2_ nanoparticles improves the PD resistance and events as well as total, mean and maximum charge magnitudes of palm oil [20]. TiO_2_ and SiO_2_ lead to the positive improvements of PDIVs for natural ester with 7.4% and 20% increments under AC voltage [21,22]. The effect of semi-conductive nanoparticle on aged MO is not consistent, whereby the PDIVs under AC voltage fluctuate with the highest percentage of improvements at low and high moisture content are 17.2% and 27.9%, respectively [23].

Generally, surfactant is introduced in order to improve the stability of nanoparticles in dielectric insulating fluids. The improvements of the PD characteristics and PDIV of MO and vegetable oil based nanofluids are quite consistent in the presence of both nanoparticles and surfactants [19,27,28,29]. With oleic acid and under AC voltage, the presence of magnetite, silica and graphene oxide caused positive impacts on the PDIVs of MO whereby the highest percentage of increments are 50%, 59.1% and 63%, respectively [28]. The improvement of PDIV could be caused by the uniform internal electrical field distribution that is contributed by the high density of shallow electron traps produced by the nanoparticles [19,27]. However, the introduction of magnetite and graphene oxide with oleic acid in MO could cause reductions of PDIVs in the range between 6.3% and 25% under positive and negative DC voltages [28]. Another study [29] has shown that the PDIV of the natural ester-based Fe_2_O_3_ nanofluids impregnated paper in the presence of oleic acid could also decrease due to the agglomeration that is formed from large nanoparticle clusters, which act as contaminants or voids, and subsequently affecting the PD activities.

The current trend in electrical power system networks is moving towards the application of green sources such as vegetable oil. Recently, palm oil and coconut oil (CO) have been identified as possible options as alternative insulating fluids in transformers. The basic electrical properties such AC/lightning breakdown voltage, partial discharge, thermal ageing performances and dielectric performances of these oils are comparable with other types of vegetable oil; however, further improvement can be carried out through chemical modifications or introduction of nanoparticles. Nanoparticles could provide the simplest solution to improve the performances of palm oil and CO. Currently, there is a lack of research especially on the PD characteristics of palm oil and CO based nanofluids. Considering nanoparticles would affect the PD properties of palm oil and CO, it is important to carry out the investigation to determine its impact for practical applications in the future.

The main motivation of the paper is to examine the impact of Al_2_O_3_ on the PD characteristics for refined, bleached and deodorized palm oil (RBDPO) and CO with and without sodium dodecyl sulfate (SDS). Prior similar studies have shown both Al_2_O_3_ and SDS have positive effects on the AC and lightning breakdown voltages of RBDPO and CO [30,31]. The first section of the study describes the materials and preparation methods of nanofluid. Next, the PD and Photo Multiplier Tube (PMT) measurement configurations are presented. The final section presents and discusses the PDIV, PD amplitude, repetition rate and emission of light signals for RBDPO- and CO-based Al_2_O_3_ nanofluids.

## 2. Materials and Methods

### 2.1. Materials

The samples investigated in this study were two RBDPO and one CO. One sample of MO also was used as benchmark for this study. The compositions of fats as well as vitamin E and A for RBDPO and CO could be obtained in [30,31]. On the other hand, the detail composition of fatty acids for both oils were determined by gas chromatography based on MPOB test methods, p.3.4:2004 and p.3.5:2004 [32].

The type of nanoparticle used in this study was insulative nanoparticle, Al_2_O_3_. Al_2_O_3_ properties used in the study could be obtained in [30]. The colour of Al_2_O_3_ is white. The density of Al_2_O_3_ is 3.96 g/cm^3^. Surfactant, SDS was used in this study in order to achieve suspension stability and subsequently avoid sedimentation of Al_2_O_3_. The appearance colour and form of SDS are white and solid while the flash point is 170 °C [30].

### 2.2. Preparation of Nanofluids

The RBDPO and CO were subjected to filtration three times by a membrane filter. The filter has a pore size of 0.2 µm. The first part of the synthesis process for RBDPO and CO based Al_2_O_3_ nanofluids was performed without SDS according to [30]. The Al_2_O_3_ was first introduced into the oils and stirred by a Fisher Scientific Isotemp heated magnetic stirrer at speed of 800 rpm. The stirring of oils and Al_2_O_3_ were carried for 30 min. The next 30 min was given to the oils to rest at ambient temperature for 30 min. The oils were later subjected to drying process at 85 °C in an air circulating oven for 48 h.

The second part of synthesis involved with SDS, whereby it was added into the oils and stirred at speed of 800 rpm for 30 min by a magnetic stirrer according to [30]. The concentration of SDS used in the study was 50% of the volume concentration of Al_2_O_3_. It was chosen as the concentration could provide the highest improvement of AC breakdown voltages of RBDPO and CO based on a similar study in [33]. Next, Al_2_O_3_ was introduced into the oils and stirred by a magnetic stirrer at speed of 800 rpm for 30 min. Ultrasonic Homogenizer—Model 300VT was used to ensure sonicate and disperse Al_2_O_3_ and SDS in the oils for 1 h. Next, the oils were rested for 30 min at ambient temperature and later dried at 85 °C in an air circulating oven for 48 h. For both synthesis processes, the oils were rested for 24 h at ambient temperature before the measurement of PD. In total, 0.001%, 0.025%, 0.035% and 0.05% of Al_2_O_3_ were used as the volume concentration in this study.

### 2.3. Partial Discharge

**Figure 1:**
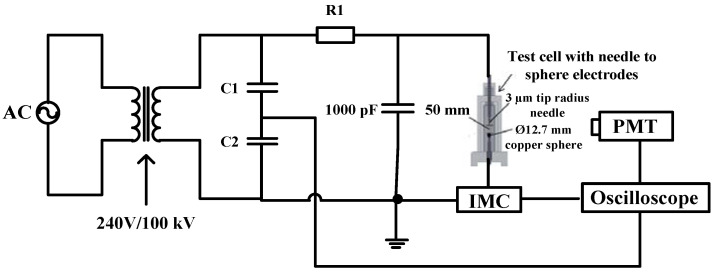
Configuration for partial discharge (PD) and photo multiplier tube (PMT) measurement.

**Figure 2:**
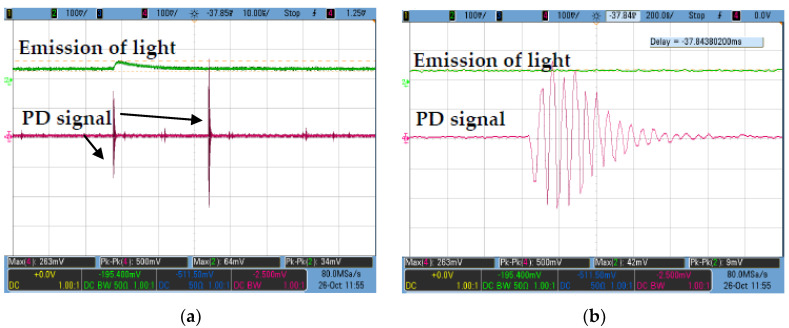
(**a**) Output voltage of the PD signal, (**b**) zoom in view for the output voltage of the PD signal waveform measured by impedance matching circuit (IMC).

The PDIV and PD measurements were performed according to IEC 61294 and IEC 60270 [34,35]. The configuration measurement for PD measurement can be seen in Figure 1. The diameter of the copper sphere electrode was 12.5 mm while the radius of needle tip was 3 µm. A 50 mm ± 1 mm gap distance was set between needle and sphere electrodes. The AC TERCO transformer with maximum voltage of 100 kV was used in this study. The oils were poured into the test cell first and allowed to stand for at least 15 min before application of the voltage. A wideband 50 Ω Impedance Matching Circuit (IMC) was used in this study to monitor the occurrences of discharges whereby it has the ability to match the load and source for maximum power transfer as well as to transform the impedances for PD measurement [36,37,38]. Next, voltage was applied to the cell, increasing at a constant rate of 1 kV/step ± 0.5 kV/step from 10 kV till a PD occurred with apparent charge equivalent to or higher than 100 pC. The output voltage of the PD signals was then recorded through channel 3 from the Agilent Technologies DS07104B oscilloscope as seen in Figure 2a. The zoom-in view of the corresponding PD waveform can be seen in Figure 2b which is similar to the output voltage signals recommended by Annex E in IEC 60270 [34].

**Figure 3:**
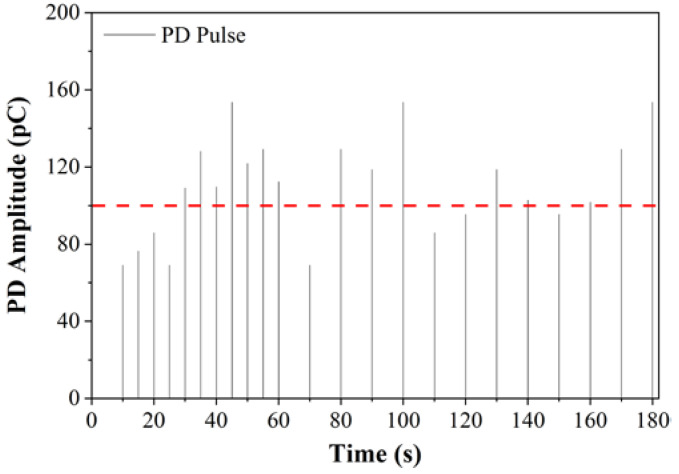
PD pulses recorded at 30 kV applied voltage for three minutes.

Previous researches used voltage duration from 1 min to 10 min to record the sufficient number of PDs [5,39]. The voltage duration needed to record a sufficient number of PDs should be long enough, but not too long to damage the needle tip by continuous discharge corrosion. In this study, the threshold value of 100 pC was used to define PDIV as per IEC 61294 [35]. According to IEC 61294, the lowest voltage level to generate a PD with an amplitude higher than 100 pC is defined as PDIV [35]. Figure 3 shows an example of all PD signals recorded in MO for duration of 3 min at 30 kV (maximum applied voltage). The maximum PD amplitude at this voltage step is about 154 pC. From the reference PDIV line in Figure 3, it is shown that 1 min is long enough to obtain the maximum PD amplitude and repetition rates. Therefore, 1 min was selected as the duration for each of the voltage steps of which the PD signal was recorded. In this study, a settling time ranging from 30 s to 1 min was given after the application of the step voltage in order to avoid disturbance signals from the system. Next, the PD signals were recorded continuously for 1 min at each of the voltage steps.

PMT can be used to record the light intensity signals [40,41]. It was used to detect faint optical signals from weakly emitting sources with a cathode luminous sensitivity 605 µA/lm and output luminous sensitivity 3632 V/lm was used to capture the PD characteristics. The photosensor module contains a metal package PMT, which has low-power consumption and low-noise amplifier that can convert the current to voltage output for ease of signal processing purpose. The PMT light oscillograms result were recorded through channel 2 from the Agilent Technologies DS07104B oscilloscope.

**Figure 4:**
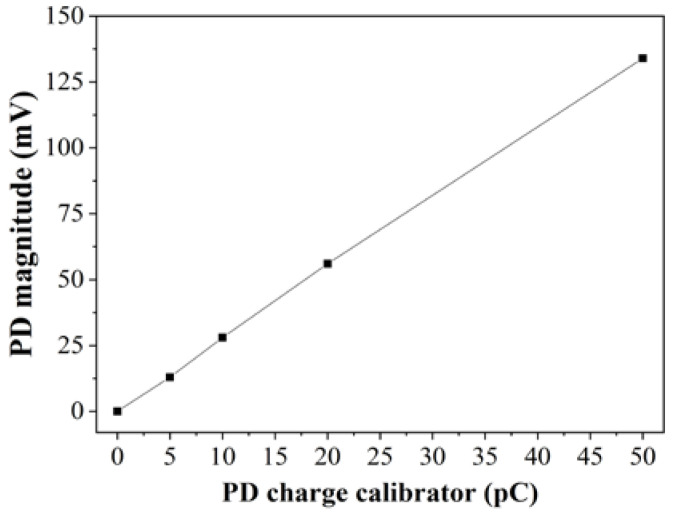
IMC reading based on charge calibrator.

It is known that the apparent charge is not equivalent to the actual amount at PD location. However, it can be measured and calibrated accordingly, and it is normally expressed in picocoulombs (pC). Apparent charge was measured through calibration of the voltage spikes against the voltage obtained from the calibration discharge into the measuring instrument. The calibration was carried out using an external JZF-10 PD calibrator detector. IMC was calibrated at four different pulse charges, which were 5 pC, 10 pC, 20 pC and 50 pC through injection at the point of measurement. The linear correlation between PD charge calibrator and measured PD magnitude were measured by the oscilloscope with r^2^ was equal to 1 as shown in Figure 4.

## 3. Results

### 3.1. RBDPO and CO Based Al_2_O_3_ Nanofluids without SDS

**Figure 5:**
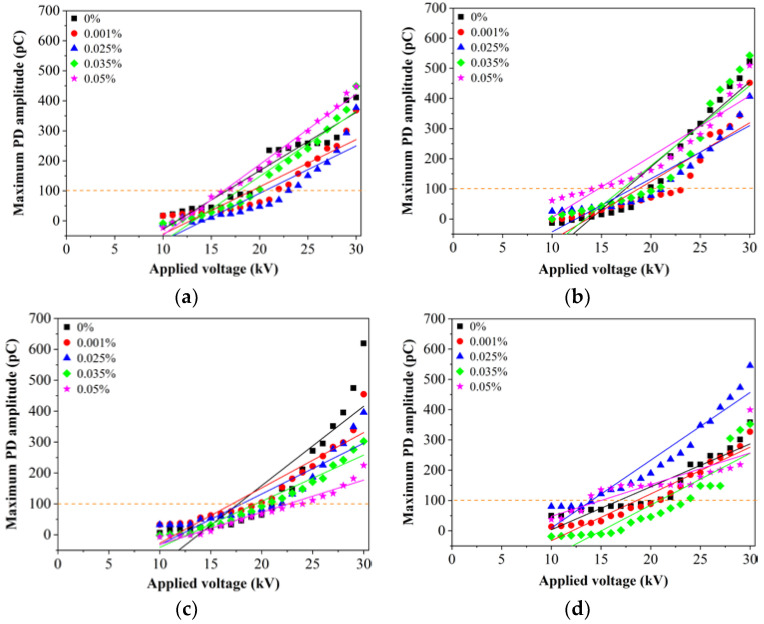
Maximum PD amplitude versus applied voltage for (**a**) RBDPOA, (**b**) RBDPOB, (**c**) CO, and (**d**) MO based Al_2_O_3_ without SDS.

Figure 5 shows the relationship between the maximum PD amplitude and applied voltage for RBDPO, CO and MO based Al_2_O_3_ nanofluids without SDS. The same approach as [1] has been applied to determine the PDIV at threshold of 100 pC based on linear fitting since the maximum amplitude and logarithm of PD repetition rate increase linearly with the increment of the applied voltage around the PDIV level as shown in Figure 5a–d. The highest percentage of PDIV increment for RBDPOA is 15% at 0.025% of Al_2_O_3_. At 0.001% of Al_2_O_3_, the PDIV of RBDPOB increases by 20%. The PDIVs of CO and MO increase by 13.6% and 14% at 0.05% and 0.035% of Al_2_O_3_. Overall, the patterns of the PD amplitudes for all types of oils show 2 distinctive features. The first pattern appears at region less than 100 pC whereby the PD amplitude increases slowly as the voltage increases. The PD amplitudes for all types of oils increase significantly as the voltage is further increased. It is shown that the introduction of Al_2_O_3_ could improve the PD amplitudes of the oils through reduction of the levels lower than base oils as the voltage is increased. At 0.001% and 0.025% of Al_2_O_3_ the PD amplitudes for RBDPOA show apparent improvements as the voltage increases from 20 kV to 27 kV as shown in Figure 5a. As the voltage increases to 30 kV, the PD amplitudes for RBDPOA are quite close but at lower levels than base RBDPOA at 0.001% and 0.025% of Al_2_O_3_. The PD amplitudes of RBDPOA do not improve at 0.035% and 0.05% of Al_2_O_3_.

A similar pattern is found at 0.05% of Al_2_O_3_ for RBDPOB except the PD amplitude increases higher than base RBDPOB as the voltage is increased from 10 kV to 22 kV as shown in Figure 5b. Apparent improvements of PD amplitudes for CO occur as the voltage increases higher than 25 kV as shown in Figure 5c. Overall, 0.05% of Al_2_O_3_ provides the highest improvements of PD amplitude of CO followed by 0.035%, 0.025% and 0.001% of Al_2_O_3_. There are improvements of PD amplitudes of MO as the Al_2_O_3_ is introduced as shown in Figure 5d. At 0.035% of Al_2_O_3_, the PD amplitude of MO maintain lower than base MO as the voltage increases from 10 kV to 27 kV. The PD amplitudes of MO do not show any apparent improvements at 0.001% and 0.05% of Al_2_O_3_. At 0.025% of Al_2_O_3_, the MO could cause the PD amplitude to increase higher than base MO at all voltage levels.

**Figure 6:**
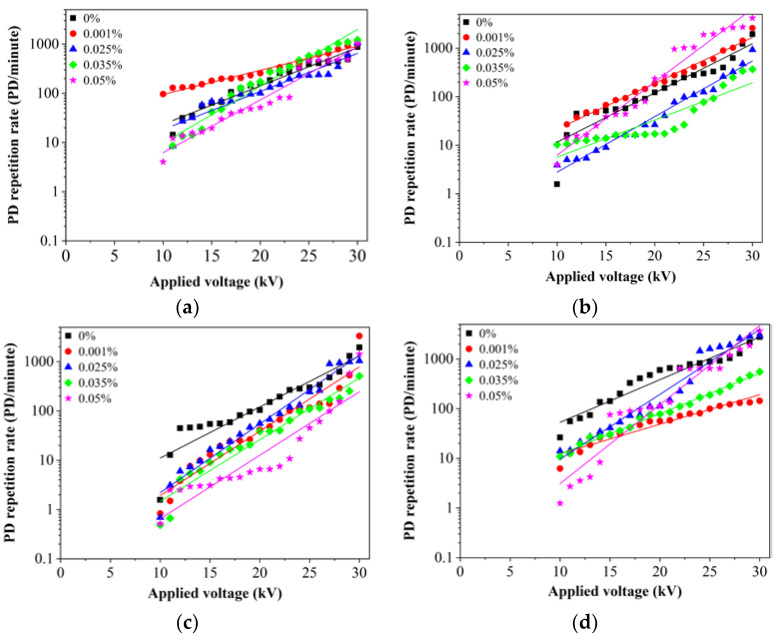
PD repetition rate versus applied voltage for (**a**) RBDPOA, (**b**) RBDPOB, (**c**) CO and (**d**) MO based Al_2_O_3_ without SDS.

Since the charge of the first detectable PD is quite small, it is practical to obtain the repetition rate as additional information to characterize the discharge activities of the oils. The PD repetition rate is characterized as the total number of PD pulses measured within chosen time interval which is normally 1 min. During the experiment, the applied voltage was increased step by step (1 kV/step) with each step lasting for 1 min. The number of PD pulses was counted in each step whereby the PD repetition rate (PD number/minute) was determined based on the overall PD number over the time duration. To date, there is still no acceptance/threshold limit for repetition rate of PD. At 0.05% of Al_2_O_3_, the PD repetition rate of RBDPOA maintains lower than base RBDPOA as the voltage increases from 10 kV to 23 kV as shown in Figure 6a. It is found that the introduction of Al_2_O_3_ could improve the PD repetition rates of the oils through reduction of the rates lower than base oils as the voltage is increased. The PD repetition rate of RBDPOA does not show clear improvement at 0.001% of Al_2_O_3_. The PD repetition rate of RBDPOA, shows a slight improvement as the voltage increases from 16 kV to 27 kV at 0.025% of Al_2_O_3_. At 0.035% of Al_2_O_3_, the introduction of 0.035% of Al_2_O_3_ could cause the PD repetition rate of RBDPOA to increase higher than base RBDPOA as the voltage is increased higher than 21 kV. Apparent improvements of PD repetition rates for RBDPOB are observed as the voltage is increased from 11 kV to 30 kV at 0.025% and 0.035% of Al_2_O_3_ as shown in Figure 6b. At 0.001% of Al_2_O_3,_ the PD repetition rate of RBDPOB maintains at higher level than base RBDPOB at all applied voltages. The PD repetition rate of RBDPOB maintains lower than that base RBDPOB as the voltage increases from 11 kV to 19 kV at 0.05% of Al_2_O_3_. The introduction of Al_2_O_3_ could improve the PD repetition rates of CO at all volume concentrations as the voltage is increased. The PD repetition rate of CO could increase higher than base CO at voltage levels of 27 kV and 28 kV for 0.025% of Al_2_O_3_ and at voltage level of 30 kV for 0.001% of Al_2_O_3_ as shown in Figure 6c. There are improvements of PD repetition rates of MO as the Al_2_O_3_ is introduced as shown in Figure 6d. Only 0.025% of Al_2_O_3_ could cause the PD repetition rate of MO to increase higher than base MO at voltage levels between 24 kV and 30 kV.

### 3.2. RBDPO, CO and MO Based Al_2_O_3_ Nanofluids with SDS

**Figure 7:**
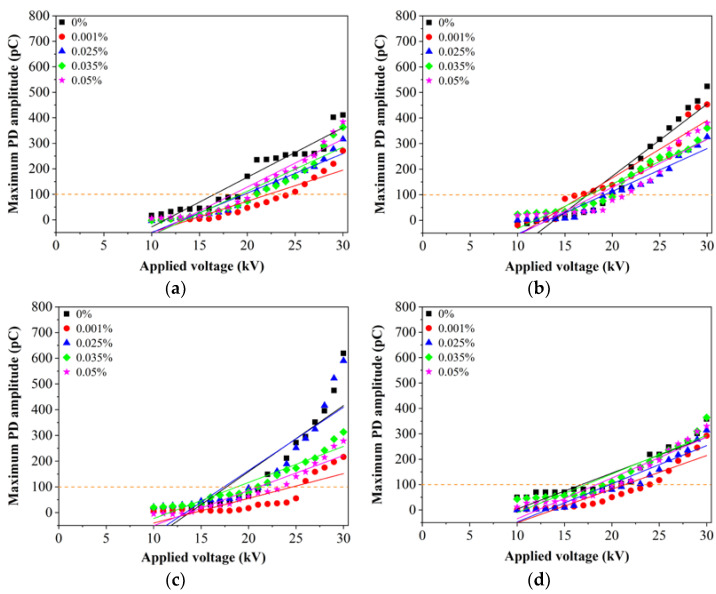
Maximum PD amplitude versus applied voltage for (**a**) RBDPOA, (**b**) RBDPOB, (**c**) CO and (**d**) MO based Al_2_O_3_ with SDS.

There is a significant effect of SDS on the PDIV of both RBDPO, CO and MO as shown in Figure 7. The highest percentages of PDIV increments for RBDPOA, CO and MO are 25%, 18% and 14% at 0.001% of Al_2_O_3_. On the other hand, RBDPOB experiences up to 10% increment of PDIV at 0.05% of Al_2_O_3_. Generally, the PD amplitudes of RBDPOA, RBDPOB, CO and MO improve with the introduction of SDS and Al_2_O_3_. The PD amplitudes for RBDPOA show apparent improvements as the voltage increases higher than 20 kV at 0.001% and 0.025% of Al_2_O_3_ as shown in Figure 7a. At 0.035% and 0.05% of Al_2_O_3_, the PD amplitudes for RBDPOA are quite close but at lower levels than that base RBDPOA as the voltage increases to 30 kV. The PD amplitudes for RBDPOB improve as the voltage increases higher than 22 kV all volume concentrations of Al_2_O_3_ as shown in Figure 7b. Clear improvement of PD amplitude for CO is found as the applied voltage increases higher than 19 kV at 0.001% of Al_2_O_3_. At 0.035% and 0.05% of Al_2_O_3_ the PD amplitudes of CO improve as the voltage increases higher than 22 kV as shown in Figure 7c. The PD amplitudes of CO maintain quite close to base CO for all voltage levels at 0.025% of Al_2_O_3_. The PD amplitudes of MO show clear improvements with the increment of the voltage at 0.001% and 0.025% of Al_2_O_3_ as shown in Figure 7d. At 0.035% of Al_2_O_3_, the PD amplitude of MO could lead to higher PD amplitude than base MO as the voltage is increased from 19 kV to 22 kV.

**Figure 8:**
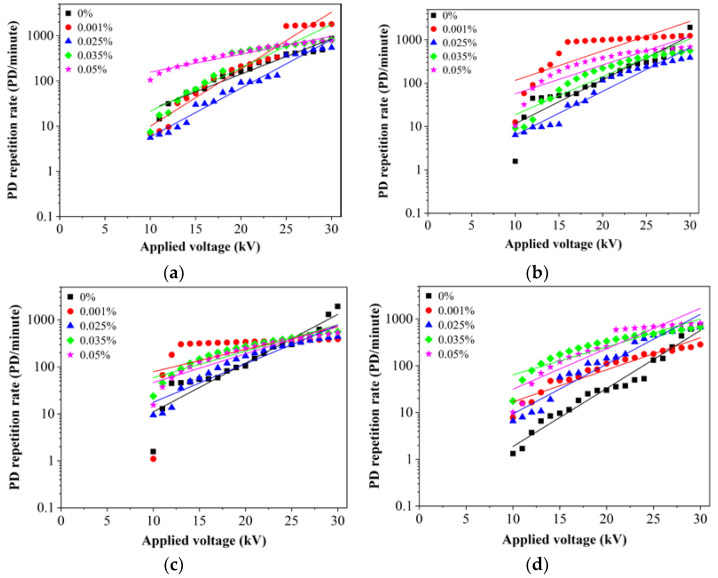
PD repetition rate versus applied voltage for (**a**) RBDPOA, (**b**) RBDPOB, (**c**) CO and (**d**) MO based Al_2_O_3_ with SDS.

In general, the presence of SDS could increase the PD repetition rates of the oils higher than base oils as the voltage is increased. At 0.001% of Al_2_O_3_, the PD repetition rate of RBDPOA is higher than base RBDPOA as the voltage increases higher than 25 kV. As shown in Figure 8a, the PD repetition rate of RBDPOA only improves as 0.025% of Al_2_O_3_ is introduced at all applied voltages. At 0.05% of Al_2_O_3_, RBDPOA could cause the PD repetition rate to increase higher than base RBDPOA as the voltage increases to 29 kV. A similar pattern is observed at 0.035% and 0.05% of Al_2_O_3_ for RBDPOB except the PD repetition rates could be lower than base RBDPOB as the voltage is increased between 29 kV and 30 kV. The PD repetition rate of RBDPOB slightly improves as the voltage increases to 30 kV at 0.025% of Al_2_O_3_ as shown in Figure 8b. Apparent improvement of PD repetition rates of CO occurs as the voltage is increased higher than 28 kV at all volume concentration of Al_2_O_3_ as shown in Figure 8c. The presence of most volume concentrations of Al_2_O_3_ and SDS do not improve PD repetition rates of MO as shown in Figure 8d. The PD repetition rates of MO are higher than base MO at all voltage levels.

## 4. Discussion

Generally, PD occurs once the electric field stress attains certain level which is known as the inception [20]. It is used as an important parameter to determine the condition of dielectric insulating fluids. PDIV is usually defined as the applied voltage whereby the first PD occurs of which the apparent charges equivalent to or higher than 100 pC. According to the findings in this study, it is shown that Al_2_O_3_ could improve the performance of PDIV of most RBDPO, CO and MO either with or without SDS as shown in Table 1. The improvements of PDIV of the all oils occur at no explicit volume concentrations of Al_2_O_3_. Without SDS, the highest improvement of PDIV for RBDPOA, RBDPOB, CO and MO are 15.6%, 7.5%, 27.7% and 24.2%, respectively. According to IEC 61294, PDIV and PD characteristics measurements are mainly influenced by electrode configuration, liquid nature, gap distance, initial electron generation, charge build-up through the oil gap and applied voltage [35,42]. Unlike AC breakdown voltage, the PDIV is measured using needle electrodes which is relatively independent to the contaminants such as moisture and particles [35,43]. This is because critical high field conditions in PD occurs within only a very small volume of the oils [35]. With nanoparticles such as Al_2_O_3_ in the oils, the space and electric field distribution changes affect the mobility of electrons and ionization occurrence in the oils of which electrons are generated, thus enhancing the insulation performances [20,44]. Localized PD occurs as a result of local intensity of the electric field at the ionization level and applied voltage [26]. The discharge is therefore suppressed whereby higher applied voltage is required to cause PDIVs in RBDPO, CO and MO [44,45]. The improvements of the PDIVs for RBDPO, CO and MO based Al_2_O_3_ nanofluids are also quite similar with a previous study [42].

The presence of SDS does further improve the PDIV of RBDPOA based Al_2_O_3_ nanofluids. SDS provides the highest improvement of PDIV of RBDPOA based Al_2_O_3_ nanofluids with 34% enhancement at 0.001% of Al_2_O_3_. The highest improvement of the PDIV of CO-based Al_2_O_3_ nanofluids is found with the introduction of SDS, where the highest percentage of improvement is 39.3% as compared to without SDS. The interaction between nanoparticles and surfactants contributed to the stability of the oil samples. A stable nanofluid is obtained once the repulsive force of the electrical double layer surpasses the Van der Waals force, which prevents the clustering of the nanoparticles [17,46]. The improvements of PDIVs for the RBDPO, CO and MO based Al_2_O_3_ nanofluids with SDS can be seen in Table 1. The negatively charged head of SDS could change the surface charges of Al_2_O_3_ and form repulsion forces among the nanoparticles. The agglomeration of nanoparticles therefore could be minimized by the act of the repulsion forces and lead to the improvements of the PDIVs for RBDPO, CO and MO [47]. Moreover, it is found that the differences on the nanoparticles dispersion between with and without SDS are not significant to cause agglomeration based on FTIR and particle size distribution [30].

The PD amplitude are among the important aspect for early detection of insulating fluid degradation and assessment of manufactured product or equipment performances. In this study, the maximum PD amplitudes of the RBDPO and MO increase linearly with the increment of the applied voltage close to the PDIV level. It is known that the charge magnitudes increase with the increment of the applied voltage as a result of the electric field enhancement, which further promotes the ionization process and increases the free electron production rate [20]. Previous studies have shown that the introduction of nanoparticles and surfactants lead to the reduction of PD amplitude as compared to the base oil [18,19,20]. A similar pattern is observed for RBDPO and CO whereby the Al_2_O_3_ without SDS leads to the decrement of PD amplitudes at most volume of concentrations and applied voltages. For both RBDPO, apparent decrements of PD amplitudes occur at low volume concentration of Al_2_O_3_. For CO, the decrement of PD amplitude is found at high volume concentration of Al_2_O_3_. The introduction of SDS leads to further decrement of the PD amplitudes for both RBDPO and CO at all volume concentration of Al_2_O_3_ and voltage levels. For MO, the improvement of the PD amplitude is not apparent with the introduction of Al_2_O_3_ with and without SDS. It was found that the PD amplitude of both MO could further increase as Al_2_O_3_ is introduced. The introduction of SDS in MO based Al_2_O_3_ does not further affect the decrement of PD amplitude.

The PD repetition rate is more susceptible to additives and contaminants as compared to PDIV and PD magnitude [48]. It is possible that the introduction of nanoparticles and surfactants could adsorb the additives and contaminants on its surface and subsequently reduce the occurrence of PD within a given time interval for the oils under study [18]. Without SDS, the PD repetition rates of RBDPO, CO and MO based Al_2_O_3_ nanofluids are lower than base oil as the voltage is increased. The PD repetition rate of RBDPOA decreases at 0.05% of Al_2_O_3_ while for RBDPOB, it decreases at 0.025% of Al_2_O_3_. As the voltage increases, the PD repetition rates of CO and MO decrease at all volume concentrations of Al_2_O_3_. With SDS, the PD repetition rates of RBDPO, CO and MO are higher than base oils at all volume concentrations of Al_2_O_3_ and voltage levels except PD repetition rates for RBDPOA and RBDPOB slightly decreases at 0.025% of Al_2_O_3_. The PD repetition rates of RBDPOB do not cause any improvements patterns at all volume concentrations of Al_2_O_3_.

The improvements of PD characteristics of nanofluids under study is possibly due to the charge trapping process that is contributed by the electric double layer formed around the nanoparticles and the oils [42]. As the nanoparticles come in contact with the oil, it will attract ions and create a strong bound layer close to their surfaces which is known as stern layer [42]. A diffuse layer is formed next to the stern layer whereby the number of ions decreases until falls to zero in the free oil. Both layers will create the electric double layer [42]. The electric double layer could trap the charges, which delays its build-up through the oil gap. In addition, the event could limit the impact of the charges on the consecutive of PD event. As a result, high electric fields are needed to initiate PD, which decrease the PD amplitude and repetition rate and concurrently increase the PDIV levels of the nanofluids under study. The thickness of electric double layer depends on the isoelectric point of nanoparticles and the pH value of oil. Surfactants can increase the thickness of electric double layer [42]. Consequently, the charge trapping capability increases which further delays the charge build-up between the oil gap, and thus further improve the PD characteristics through reduction of PD amplitude and repetition rate along with the increment of PDIVs of the nanofluids under study [42].

**Figure 9:**
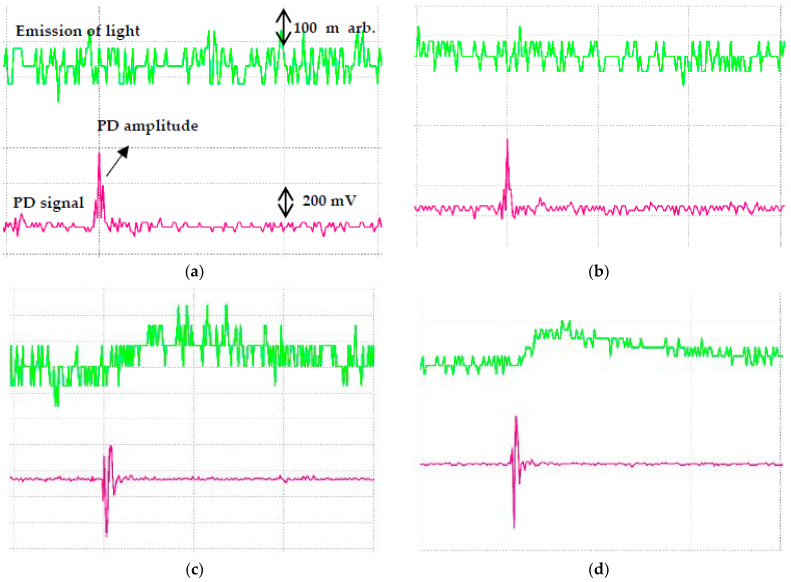

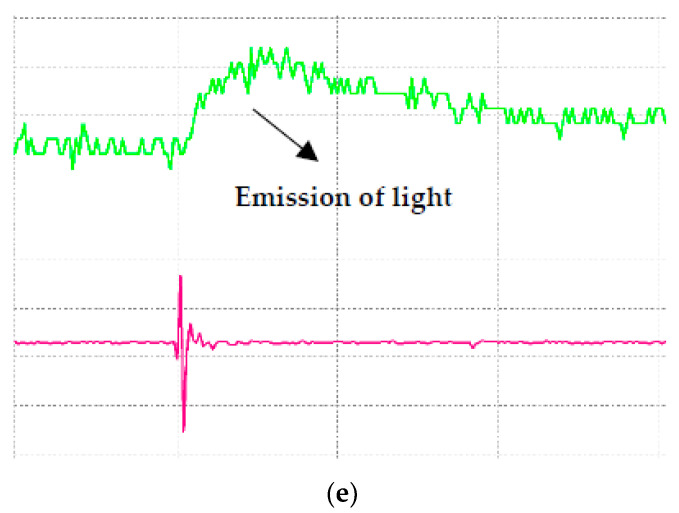
PD signal (IMC) and emission of light signals (PMT) for RBDPOA at (**a**) 10 kV, (**b**) 15 kV, (**c**) 20 kV, (**d**) 25 kV and (**e**) 30 kV applied voltage.

**Figure 10:**
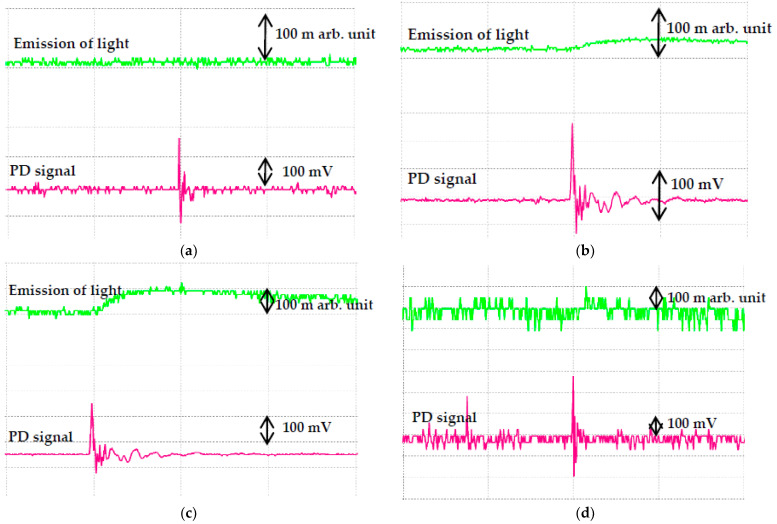
Emission of light signals (PMT) and PD signals (IMC) for (**a**) RBDPOA, (**b**) RBDPOB, (**c**) CO and (**d**) MO at 30 kV applied voltage.

**Figure 11:**
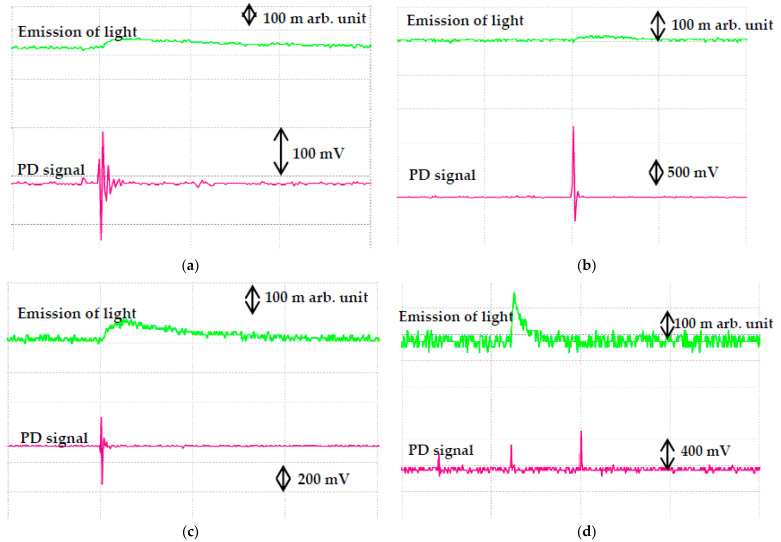
Emission of light signals (PMT) and PD signals (IMC) for (**a**) RBDPOA, (**b**) RBDPOB, (**c**) CO and (**d**) MO based Al_2_O_3_ without SDS at 30 kV applied voltage.

**Figure 12:**
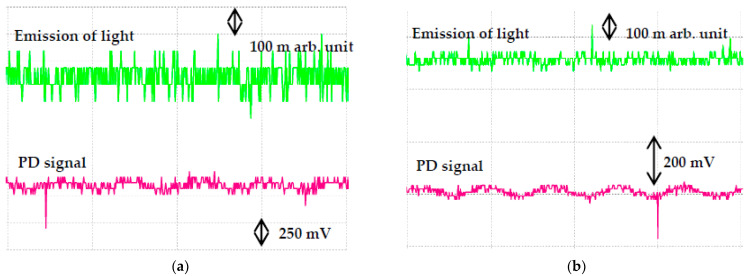

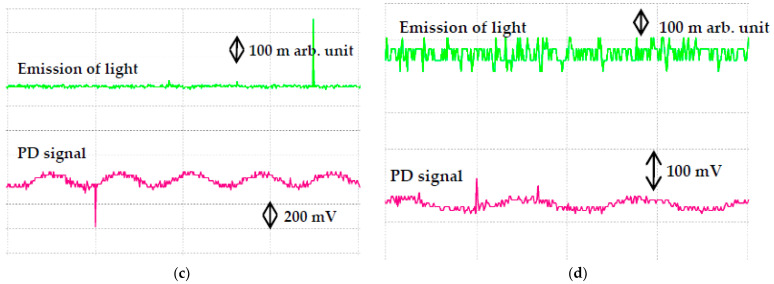
Emission of light signals (PMT) and PD signals (IMC) for (**a**) RBDPOA, (**b**) RBDPOB, (**c**) CO and (**d**) MO based Al_2_O_3_ with SDS at 30 kV applied voltage.

Based on IEC 60270, the PD measurement could be carried out through optical observation, acoustical detection, emission of light signals, sound, heat and chemical reactions methods [34]. This method is a subsequent observation of the effects of any discharges in the oils. Real time emission of light signals can be carried out using PMT to capture the PD activities. It is shown that the emitted light signals of RBDPOA that are captured by PMT correspond to the PD and voltage signals that are measured by IMC as shown in Figure 9. The emission of light signals can be emitted at higher applied voltage with different PD activities. With the increment of the voltage level, the repetition rate of the light pulse does not increase; however, the amplitude tends to increase. The emission of light signals become more intense and stronger in term of larger magnitude and higher repetition rate as the voltage level is increased. Figure 10, Figure 11 and Figure 12 show the emission of light and PD signals of RBDPO, CO and MO based Al_2_O_3_ nanofluids at applied voltage 30 kV corresponding to the volume concentration whereby the highest percentage of PDIV increment is observed. Generally, there are two types of observations between emission of light signals and PD activities for both RBDPO and MO are found in this study. The first observation occurs once the emission of light and PD signals appears simultaneously while the second observation is vice versa. The emission of light signals for base RBDPOA and MO do not appear as the PD occurs. The emission of light signal for base MO is more intense as compared to base RBDPO and CO as shown in Figure 10. In MO, the presence of aromatic molecules help to provide a stronger light emission, while the emission of light signals in RBDPO and CO are weaker as compared to MO [49]. As compared to base oils, the light emitted signals could appear simultaneously with the PD signals for RBDPO, CO and MO in the presence of Al_2_O_3_. Like base oils, emission of light signals for both MO based Al_2_O_3_ are more intense and have higher amplitudes as compared to RBDPO and CO based Al_2_O_3_. With SDS, the emission of light and PD signals of RBDPO, CO and MO become more intense and gradually stronger with larger amplitudes as compared without SDS. Similar patterns are found as base oils whereby for RBDPOA and MO with presence of SDS where there is no emission of light signal even though the PD signals appear as shown in Figure 12a,d. The higher repetition of emission of light signals tends to show in RBDPOB even though there is no PD signal as shown in Figure 12b. With SDS, the emission of light and PD signals of CO become gradually stronger at high amplitudes. The high repetition of emission of light signals tends to show in both MO even though there is no PD signal as shown in Figure 12d. Overall, the emission of light signals of RBDPO, CO and MO based Al_2_O_3_ nanofluids show inconsistent patterns whereby it fluctuate as the Al_2_O_3_ is introduced.

There are clear reduction patterns of average emission of light signals for RBDPOA, RBDPOB and CO as the Al_2_O_3_ is introduced as shown in Table 2. The lowest average emission of light signal for RBDPOA occurs at 0.05% of Al_2_O_3_. For RBDPOB and CO, the lowest average emission of light signals is at 0.035% of Al_2_O_3_. On the other hand, the average emission of light signal of MO fluctuates at a higher level than base MO with the increment of the volume concentration of Al_2_O_3_. With SDS, the average emission of light signal for RBDPOA fluctuates at higher level than base RBDPOA with the increment of the volume concentration of Al_2_O_3_. For CO, the average emission of light signal shows an increment pattern with the increment of the volume concentration of Al_2_O_3_. However, it was noticed that the average emission light signals for RBDPOB and CO decrease at lower level than base RBDPOB and CO at 0.001% of Al_2_O_3_. On the other hand, the average emission of light signal of MO continues to increase as the volume concentration of Al_2_O_3_ increases.

Since the fatty acid compositions of both RBDPO are almost similar, the differences on the patterns of PDIV, PD amplitude and repetition rate between RBDPOA and RBDPOB based Al_2_O_3_ nanofluids are possibly affected by the presence of vitamin E. The aggregation process of the surfactants could be facilitated by vitamin E. It is due to the hydrophobic interaction of α-tocopherol molecules in vitamin E with the alkyl chain of the surfactants. Instead of getting adsorbed to the Al_2_O_3_ surface, the surfactants form an aggregation with the vitamin E molecules [50,51]. On the other hand, there is no apparent effect of vitamin A on the PDIV and PD activities of RBDPOA.

## 5. Conclusions

The PDIV and PD activities generated by needle-sphere electrode configuration under AC voltage for RBDPO and CO based Al_2_O_3_ with and without SDS are experimentally measured. The PDIVs of RBDPOA, RBDPOB, CO and MO are slightly improved with Al_2_O_3_ with or without SDS. Based on the finding in this study, the PDIVs of both RBDPO and CO are comparable to MO. The SDS could promote further improvement of PDIVs for all samples. SDS could provide 34% and 39% improvement on the PDIVs of RBDPOA and CO based Al_2_O_3_ nanofluids. The PD amplitude increases linearly with the increment of voltage levels. The presence of SDS could further improve the PD amplitudes of RBDPO and CO at most volume concentrations of Al_2_O_3_. Without SDS, the highest improvement of PD amplitudes for RBDPO and CO are 22% and 64%. The PD repetition rates of RBDPO and CO can be enhanced up to 81% and 74% respectively. With SDS, the PD amplitude and repetition rate of RBDPO improve by 38% and 80% while for CO, it can be enhanced up to 65% and 80%, respectively. For MO, the improvement of PD amplitude and repetition rate are around 18% and 95% regardless with and without SDS. Generally, it is found that without SDS, the average emission of light signals for RBDPOA, RBDPOB and CO decrease by 26%, 75% and 65% with the improvement of PD characteristics, but both events do not correlate at the same volume concentration of Al_2_O_3_. Meanwhile, the presence of SDS leads to the increment of emitted light signal levels with the improvement of PD characteristics. For MO, there is no correlation can be made between emission of light signals and PD characteristics either with or without SDS. Current research shows that there is no apparent conclusion that can be made on the suitable concentration of Al_2_O_3_ nanoparticles that can provide the optimum improvement of PDIV, PD amplitudes and PD repetition rates of RBDPO, CO and MO. The current study shows that the introduction of Al_2_O_3_ and SDS has a promising impact on the partial discharge properties of either RBDPO or CO. However, further study is required in order to evaluate other important properties such the temperature stability of RBDPO and MO based Al_2_O_3_ nanofluids in the presence of SDS for future practical application in transformers.

## Figures and Tables

**Table 1:** Percentages of PDIV increment or decrement for RBDPO, CO and MO Al_2_O_3_ based nanofluids.

Samples	Volume of Concentration (%)	Percentage Increment or Decrement (%)	
		Without SDS	With SDS
RBDPOA	0.001	+15.6	+34
	0.025	+23.9	+18.8
	0.035	+7.4	+16.4
	0.05	−1.8	+11.6
RBDPOB	0.001	+7.5	−2.2
	0.025	+3.7	+10.2
	0.035	−1.8	+0.8
	0.05	−15.8	+5.9
CO	0.001	−3.2	+39.3
	0.025	+2	−1.6
	0.035	+9.7	+6.2
	0.05	+27.7	+19
MO	0.001	+10.6	+27
	0.025	−16.4	−17.6
	0.035	+24.2	+2.6
	0.05	−10.8	+9.4

* The percentage was calculated based on PDIV values of base oil; * ‘+’: increment and ‘−‘: decrement.

**Table 2:** Average emission of light signals of RBDPO, CO and MO based Al_2_O_3_ nanofluids at 30 kV applied voltage.

Samples	Volume of Concentration (%)	Average Emission of Light Signals (m arb. Unit)	
		Without SDS	With SDS
RBDPOA	0	60	60
	0.001	77.6	109.9
	0.025	52.3	154.1
	0.035	53.6	111.6
	0.05	44.6	219.4
RBDPOB	0	96	96
	0.001	82.2	49
	0.025	24.1	172
	0.035	24	49.7
	0.05	45.6	152.2
CO	0	105.4	105.4
	0.001	61.3	78.5
	0.025	71	140.8
	0.035	36.8	163.5
	0.05	264.2	280.3
MO	0	56.7	56.7
	0.001	150.2	120.8
	0.025	328.8	128.9
	0.035	224.8	348.4
	0.05	102.4	428

## References

[1] Wang X. (2011). Partial Discharge Behaviours and Breakdown Mechanisms of Ester Transformer Liquids under AC Stress. Ph.D. Thesis.

[2] Azmi K., Ishak D., Kamarol M., Zuhairi A. Comparison of Partial Discharge Behavior in Mineral Oil and PFAE under Influence of Spherical Metal Particle. Proceedings of the International Conference on High Voltage Engineering and Power System.

[3] Lesaint O. Streamers in Liquids: Relation with Practical High Voltage Insulation and Testing of Liquids. Proceedings of the IEEE International Conference Dielectirc Liquids.

[4] Natrras D.A. (1988). Partial Discharge Measurement and Interpretation. IEEE Electr. Insul. Mag..

[5] Forster E.O. (1993). Partial Discharge and Streamers in Liquid Dielectrics—The Significance of the Inception Voltage. IEEE Trans. Dielectr. Electr. Insul..

[6] Cavallini A., Montanari G.C., Ciani F. Analysis of Partial Discharge Phenomena in Paper-oil Insulation Systems as a Basis for Risk Assessment Evaluation. Proceedings of the IEEE International Conference on Dielectric Liquids.

[7] Yu W., Xie H. (2012). A Review on Nanofluids: Preparation, Stability Mechanism and Applications. J. Nanomater..

[8] Murshed S.M.S., Leong K.C., Yang C. (2005). Enhanced Thermal Conductivity of TiO_2_—Water based Nanofluids. Inter. J. Therm. Sci..

[9] Daungthongsuk W., Wongwises S. (2007). A Critical Review of Convective Heat Transfer of Nanofluids. Renew. Sustain. Energy Rev..

[10] Utomo A., Poth H., Robbins P., Pacek A. (2012). Experimental and Theoretical Studies of Thermal Conductivity, Viscosity and Heat Transfer Coefficient of Titania and Alumina Nanofluids. Int. J. Heat Mass Transf..

[11] Gkountas A.A., Benos T.L., Sofiadis N.G., Sarris E.I. (2021). A Printed-Circuit Heat Exchanger Consideration by Exploiting an Al_2_O_3_-water Nanofluid: Effect of the Nanoparticles Interfacial Layer on Heat Transfer. Therm. Sci. Eng. Prog..

[12] Attalla M., Maghrabie H.M. (2019). An Experimental Study on Heat Transfer and Fluid Flow of Rough Plate Heat Exchanger using Al_2_O_3_/Water Nanofluid. J. Therm. Energy Gener. Transp. Storage Convers..

[13] Gkountas A.A., Benos T.L., Nikas K.S., Sarris I.E. (2020). Heat Transfer Improvement by an Al_2_O_3_-water nanofluid coolant in Printed-Circuit Heat Exchangers of Supercritical CO_2_ Brayton Cycle. Therm. Sci. Eng. Prog..

[14] Benos L., Karvelas E.G., Sarris I.E. (2019). A Theoretical Model for the Magnetohydrodynamic Natural Convection of A CNT-Water Nanofluid Incorporating a Renovated Hamilton-Crosser Model. Int. J. Heat Mass Transf..

[15] Ranjbarzadeh R., Isfahani A.H.M., Hojaji M. (2018). Experimental Investigation of Heat Transfer and Friction Coefficient of the Water/Graphene Oxide Nanofluid in a Pipe Containing Twisted Tape Inserts Under Air Cross-Flow. J. Therm. Energy Gener. Transp. Storage Convers..

[16] Xuan Y., Li Q. (2000). Heat Transfer Enhancement of Nanofluids. Int. J. Heat Fluid Flow.

[17] Rafiq M., Lv Y.Z., Li C. (2016). A Review on Properties, Opportunities and Challenges of Transformer Oil-based Nanofluids. J. Nanomater..

[18] Jin H.F., Morshuis P., Rodrigo Mor A., Smit J.J., Andritsh T. (2015). Partial Discharge Behavior of Mineral Oil based Nanofluids. IEEE Trans. Dielectr. Electr. Insul..

[19] Du Y.F., Lv Y.Z., Li C., Chen M., Zhong Y., Zhou J., Li X.X., Zhou Y. (2012). Effect of Semiconductive Nanoparticles on Insulating Performances of Transformer Oil. IEEE Trans. Dielectr. Electr. Insul..

[20] Makmud M.Z.H., Alias H.A., Chee C.Y., Dabbak S.Z.A. (2019). Partial Discharge in Nanofluid Insulation Material with Conductive and Semiconductive Nanoaparticles. Materials.

[21] Zhong Y., Lv Y., Li C., Du Y., Chen M., Zhang S., Zhou Y., Chen L. (2013). Insulating Properties and Charge Characteristics of Natural Ester Fluid Modified by TiO_2_ Semiconductive Nanoparticles. IEEE Trans. Dielectr. Electr. Insul..

[22] Prasad D., Chandrasekar S. (2017). Effect of Nano-SiO_2_ Particles on Partial Discharge Signal Characteristics of FR3 Transformer Oil. J. Adv. Chem..

[23] Lv Y.Z., Du Y.F., Zhou J.Q., Li X.X., Chen M.T., Li C.R., Wang G.L. (2012). Nanoparticle Effect on Electrical Properties of Aged Mineral Oil based Nanofluids. CIGRE.

[24] Kurimsky J., Rajnak M., Cimbala R., Paulovicova K., Rozynek Z., Kopcansky P., Timko M. (2021). Electrical Discharges in Ferrofluids based on Mineral Oil and Novel Gas-to-Liquid Oil. J. Mol. Liq..

[25] Swati K., Sarathi R., Yadav K.S., Taylor N., Edin H. (2018). Corona Discharge Activity in Nanoparticle Dispersed Transformer Oil under Composite Voltages. IEEE Trans. Dielectr. Electr. Insul..

[26] Swati K., Yadav K.S., Sarathi R., Vinu R., Danikas M.G. (2017). Understanding corona discharge activity in titania nanoparticles dispersed in transformer oil under ac and dc voltages. IEEE Trans. Dielectr. Electr. Insul..

[27] Du Y., Lv Y., Li C., Chen M., Zhou J., Li X., Zhou Y., Tu Y. (2011). Effect of Electron Shallow Trap on Breakdown Performance of Transformer Oil-based Nanofluids. J. Appl. Phys..

[28] Cavallini A., Karthik R., Negri F. (2015). The Effect of Magnetite, Graphene Oxide and Silicone Oxide Nanoparticles on Dielectric Withstand Characteristics of Mineral Oil. IEEE Trans. Dielectr. Electr. Insul..

[29] Peppas G.D., Charalampakos V.P., Pyrgioti E.C., Tsovilis T., Politis Z., Gonos I.F. Partial Discharge Study of Ultrastable Colloidal Nanofluid Impregnated Paper. Proceedings of the 20th International Symposium on High Voltage Engineering.

[30] Mohamad N.A., Azis N., Jasni J., Ab Kadir M.Z.A., Yunus R., Yaakub Z. (2020). Effect of Surfactants on the Lightning Breakdown Voltage of Palm Oil and Coconut Oil based Al_2_O_3_ Nanofluids. Nanotechnology.

[31] Mohamad N.A., Azis N., Jasni J., Ab Kadir M.Z.A., Yunus R., Yaakub Z. (2019). Impact of Fe_3_O_4_, CuO and Al_2_O_3_ on the AC Breakdown Voltage of Palm Oil and Coconut Oil in the Presence of CTAB. Energies.

[32] ISO 5508:1990 (1990). Animal and Vegetable Fats and Oils-Analysis by Gas Chromatography (GC) of Methyl-Esters of Fatty Acids.

[33] Mohamad N.A., Azis N., Jasni J., Ab Kadir M.Z.A., Yunus R., Yaakub Z. Effects of Different Types of Surfactants on AC Breakdown Voltage of Refined, Bleached and Deodorized Palm Oil based CuO Nanofluids. Proceedings of the IEEE PES Asia-Pacific Power and Energy Engineering Conference (APPEEC).

[34] IEC 60270 (2000). High-Voltage Test Techniques—Partial Discharge Measurements.

[35] IEC 61294 (1993). Insulating Liquids—Determination of the Partial Discharge Inception Voltage (PDIV)—Test Procedure.

[36] Zawawi W.M.W.A.I., Makmud M.Z.H., Arief Y.Z. (2012). A Study on the Performance of Impedance Matching Circuit in Partial Discharge Measuring System. Borneo Sci..

[37] Azmi K., Zuhairi A., Ishak D., Muhamad N.A., Kamarol M. Partial Discharge Characteristics of Spherical Metal Particle in Mineral Oil and PFAE under AC Voltage. Proceedings of the International Symposium Electrical Insulating Materials (ISEIM).

[38] Abrie P.L.D. (1985). The Design of Impedance-Matching Networks for Radio-Frequency and Microwave Amplifiers.

[39] Mazzetti C., Pompili M., Forster E.O. (1992). A Study of Partial Discharge Measurements in Dielectric Liquids. IEEE Trans. Dielectr. Electr. Insul..

[40] Liu Q., Wang Z.D. (2011). Secondary Reverse Streamer Observed in an Ester Insulating Liquid under Negative Impulse Voltage. J. Phys. D Appl. Phys..

[41] Liu Q., Wang Z.D. (2011). Streamer Characteristic and Breakdown in Synthetic and Natural Ester Transformer Liquids with Pressboard Interface under Lightning Impulse Voltage. IEEE Trans. Dielectr. Electr. Insul..

[42] Eman G.A., Mansour D.E.A., Izzularab M.A. (2020). Partial Discharge Development in Oil-based Nanofluids: Inception, Propagation and Time Transition. IEEE Access.

[43] Kurimsky J., Rajnak M., Cimbala R., Jakub Rajnic M.T., Kopcansky P. (2020). Effect of Magnetic Nanoparticles on Partial Discharges in Transformer Oil. J. Magn. Magn. Mater..

[44] Wang Q., Rafiq M., Lv Y., Li C., Yi K. (2016). Preparation of Three Types of Transformer Oil-based Nanofluids and Comparative Study on the Effect of Nanoparticle Concentrations on Insulating Property of Transformer Oil. J. Nanotech..

[45] Holtzhausen J.P., Vosloo W.L. (2008). High Voltage Engineering: Practice and Theory.

[46] Ali N., Teixeira J.A., Addali A. (2018). A Review on Nanofluids: Fabrication, Stability, and Thermophysical Properties. J. Nanomater..

[47] Simpson S., Schelfhout A., Golden C., Vafaei S. (2018). Nanofluid Thermal Conductivity and Effective Parameters. Appl. Sci..

[48] Liu Z., Liu Q., Wang Z.D., Jarman P., Krause C.H., Smith P.W.R., Gyore A. Partial Discharge Behaviour of Transformer Liquids and the Influence of Moisture Content. Proceedings of the IEEE International Conference Liquids Dielectric.

[49] Lesaint O., Massala G. (1998). Positive Streamer Propagation in Large Oil Gaps: Experimental Characterization of Propagation Modes. IEEE Trans. Dielectr. Electr. Insul..

[50] Demissie H., Duraisamy R. (2016). Effects of Electrolytes on the Surface and Micellar Characteristics of Sodium Dodecyl Sulphate Surfactant Solution. J. Sci. Innov. Res..

[51] Cieśla J., Koczańska M., Narkiewicz-Michałek J., Szymula M., Bieganowski A. (2016). The Physicochemical Properties of CTAB Solutions in the Presence of α-tocopherol. J. Mol. Liq..

